# Developing a culturally appropriate illustrated tool for the self-collection of anorectal specimens for the testing of sexually transmitted infections: lessons from Papua New Guinea

**DOI:** 10.1186/s12889-019-6506-x

**Published:** 2019-02-20

**Authors:** Angela Kelly-Hanku, Stephen Bell, Sophie Ase, Ruthy Boli-Neo, Andrew J. Vallely, Steven G. Badman, Claire E. Nightingale, Johanna Wapling

**Affiliations:** 1 Sexual and Reproductive Health Unit, Papua New Guinea Institute of Medical Research, Goroka, Papua New Guinea; 2 The Kirby Institute, UNSW Sydney, Sydney, Australia; 3 Centre for Social Research in Health, UNSW Sydney, Sydney, Australia

**Keywords:** Anorectal STIs, Papua New Guinea, Self-collection, STI testing, Culturally appropriate, Key populations

## Abstract

**Background:**

Papua New Guinea (PNG) has a high prevalence of sexually transmitted infections (STIs). There is increasing evidence that anorectal STIs are important in terms of the dual epidemics of HIV and STIs in this setting. At the time of this study, anorectal STI testing was not possible, and there was no mechanism for self-collection of anorectal specimen among at risk ‘key populations’. This paper documents the development of a culturally appropriate tool that has been used to facilitate self-collection of anorectal specimens with key populations in PNG.

**Methods:**

This qualitative study involved four focus groups conducted with a purposive sample of 35 participants, including female sex workers, men who have sex with men and transgender women in Port Moresby and Goroka in 2015. During focus groups, participants reviewed and provided critical feedback for the adaption of a previously piloted and published pictorial anorectal specimen collection tool for use with key populations in PNG.

**Results:**

The final instruction tools are presented in English language and Tok Pisin. To develop these, participants feedback resulted in six key areas of the existing instruction document being modified to ensure it was appropriate for use in PNG. These included translating complex words for sexual health issues (i.e. ‘STIs’, ‘anorectal STIs’, ‘anus’, ‘anal sex’), biomedical instruments (i.e. ‘specimen bottle’, ‘specimen packet’ and ‘swab’), and aspects of the clinical procedure (i.e. inserting the swab 3–4 cm into the anus to collect a specimen). The visual identity of the graphics was redesigned to localise the images for use in PNG.

**Conclusions:**

This paper describes the development of a culturally and linguistically appropriate tool for a biomedical and clinical intervention with key populations in PNG based around self-collection of anorectal specimens for molecular STI testing. The final tools have been used to facilitate the self-collection of anorectal specimens following a clear clinical protocol during a large bio-behavioural survey in PNG.

## Background

Unprotected heterosexual or homosexual anal intercourse poses important public health risks for the management of sexually transmitted infections (STIs). Bacterial STIs, including gonorrhoea and chlamydia, can colonise the rectum, and often go unreported, undiagnosed and untreated [1]. In many low- and middle-income settings, including Papua New Guinea (PNG), resources and facilities to perform anorectal STI screening are not available, and STI treatment guidelines are not specific for the anatomical location of the infection. There is increasing evidence that anorectal STIs are important in terms of the dual epidemics of HIV and STIs in PNG [2, 3]. Diagnosing and treating STIs, including anorectal STIs in ‘key populations’ – groups of people who are key to the dynamics of, or response to, epidemics of STIs and the human immunodeficiency virus (HIV) [4, 5] – is vital in a country’s STI and HIV response, and PNG is no exception [2, 6].

There has been substantial interest in understanding the acceptability, feasibility and performance of alternative collection methods of specimens for testing bacterial STIs [7, 8], and anal cytology among men who have sex with men [9]. Several recent studies evaluated self-collected vaginal and anorectal swabs for STI diagnosis [8, 9]. In PNG, self-collected vaginal swabs have been used in studies among women attending antenatal, sexual health and well woman clinics to diagnose STIs [10–12]. The use of self-collected samples tested using novel point of care technologies has shown comparable performance to clinician-collected samples [13]. Prior to this study, however, self-collection of anal swabs for anorectal STI testing had not occurred in PNG.

The first stage of this qualitative study documented overwhelming support for anorectal STI testing and self-collection of anorectal specimens among female sex workers, men who have sex with men and transgender women in PNG [6]. The second stage of this study involved the design of a culturally appropriate tool for self-collected anal swabs for use during a large biobehavioural survey involving these key populations in PNG [14–16]. The aim of this paper is to present the results of the development of a culturally appropriate tool needed to implement effective anorectal STI testing programs in PNG.

## Methods

A qualitative research approach was adopted to engage members of civil society organisations representing key populations – female sex workers from *Friends Frangipani,* and men who have sex with men and transgender women from *Kapul Champions* – in the development of culturally appropriate illustrated instructions to facilitate self-collection of anorectal specimens for STI testing in PNG. Four focus groups were conducted in Goroka, Eastern Highlands Province (May 2015) and Port Moresby, National Capital District (August 2015); three were with female sex workers and one was with men who have sex with men and transgender women. These sites were selected to represent two diverse regions of PNG, and because the PNG Institute of Medical Research has long-established research relationships with members of key populations in these locations.

A purposive sampling technique [17] was used to recruit participants, initiated through snowballing via prominent, well-respected members of these populations known to the research team. Inclusion criteria for participants were that they were 18 years or older, a member of a key population, willing to discuss anal sex and anal STIs, and could provide informed consent. Participants did not need to report having engaged in anal sex or have experienced an anorectal STI to participate.

**Fig. 1:**
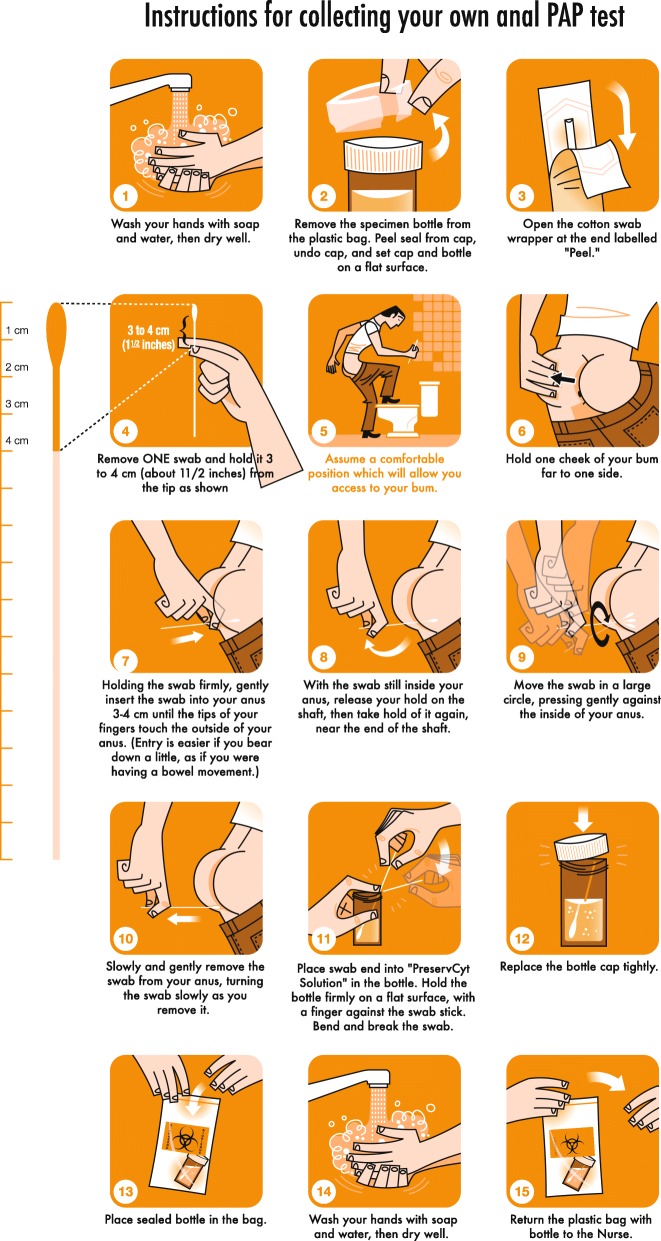
Anorectal self-collection tool adapted for key populations in Papua New Guinea*.© Luc Latulippe. * Lampinen, T.M., et al., Illustrated Instructions for Self-Collection of Anorectal Swab Specimens and Their Adequacy for Cytological Examination*. Sexually Transmitted Diseases*, 2006. 33(6):386–388. https://journals.lww.com/stdjournal/fulltext/2006/06000/Illustrated_Instructions_for_Self_Collection_of.10.aspx

During focus groups, research participants (*N* = 35) reviewed and adapted a pictorial collection tool produced for collection of anorectal swabs for anal cancer screening in the United States of America for men who have sex with men [9] (See Fig. 1). The aim of data collection was to gather research participants’ ‘emic’ (insider) perspectives to ensure that the final product was useable among key populations in PNG, and tailored to their local needs, meanings, and experiences [18].

The focus groups consisted of between 6 and 11 people and lasted up to 90 min. Each group was sex specific; with their approval, transgender women and men who have sex with men were in a single group. Focus groups enable the most appropriate investigation of community, rather than individual, perspectives [17] on sensitive sexual health issues, such as anorectal STI testing. Furthermore, group-based research fulfilled the aim of enabling representatives from key populations to debate and seek consensus on the most appropriate terminology to be used around often stigmatised sexual health and health seeking practices. Focus groups used semi-structured interview guides to explore key terms, phrases and instructions, and the visual depiction of the stages and processes in the previously published tool [9]. Figure 1 was used as an example during focus group discussions to guide discussion and create an illustrated tool for collection of anal swabs for STI testing that is appropriate for use with key populations in PNG.

The focus groups were led by PNG researchers employed within PNG Institute of Medical Research. All were fluent in *Tok Pisin* and already had extensive research experience working with and interviewing members of key populations on sensitive issues such as sexual practices. All focus groups were led by a member of the same sex. Focus group discussions were semi-structured and conducted in a safe and confidential space identified by the study participants, most often the office of a non-government organisation and in one instance the backyard of a guest lodge frequented by female sex workers.

All focus groups were digitally audio recorded, transcribed and translated from *Tok Pisin* to English. All personal identifiers were removed from the focus group transcripts and pseudonyms given to each participant. Data were subjected to rigorous thematic analysis by two researchers – AKH and SB – following a systematic process of inductive ‘open’ and ‘axial’ coding [19] using Nvivo 11 (QSR International) with no discrepancies emerging between the two coders.

The study protocol was approved by two Ethics Committees: the Papua New Guinea Institute of Medical Research Institutional Review Board (IRB1319); the Papua New Guinea National Department of Health’s Medical Research Advisory Committee (MRAC13.32). Key ethical principles adhered to throughout the investigation include voluntary participation, informed consent, confidentiality and anonymity through the use of pseudonyms.

## Results

Four focus groups were conducted with 35 participants (see Table 1). Of these participants, 21 were female sex workers and 11 identified as either a man who has sex with other men or as transgender woman.

**Table 1:** Research participant information

Focus group	Research participant group	No. of participants	Location
FGD 1	Friends Frangipani (female sex workers)	9	Goroka
FGD 2	Friends Frangipani (female sex workers)	6	Goroka
FDG 3	Kapul Champions (Men who have sex with men and Transgender women)	11	Port Moresby
FGD 4	Friends Frangipani (female sex workers)	9	Port Moresby

**Fig. 2:**
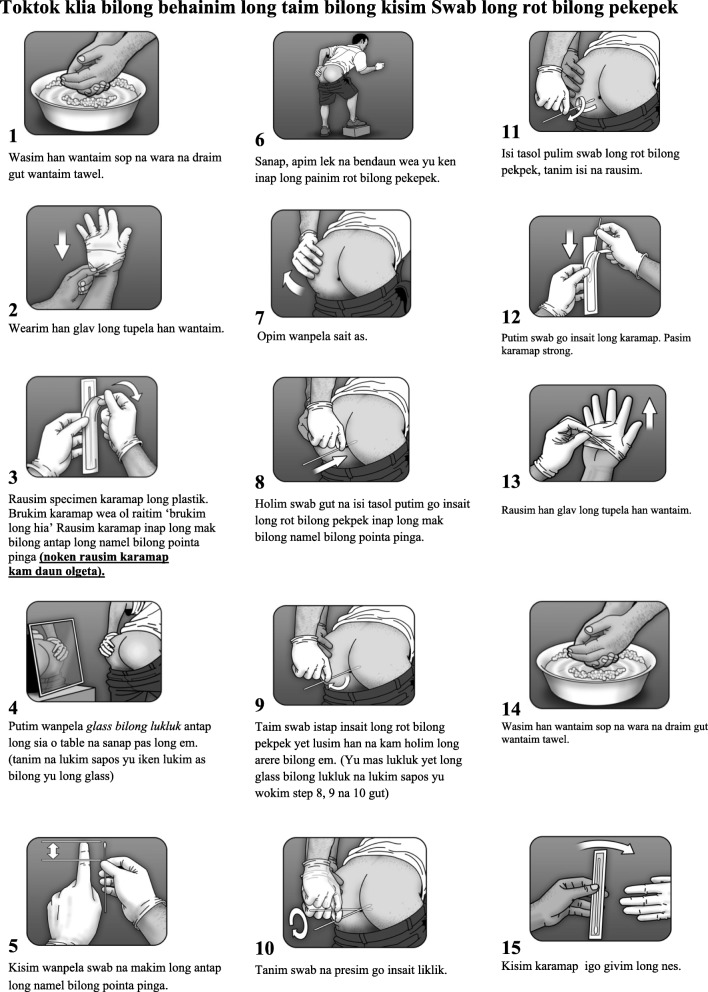
Tok Pisin version of the anorectal self-collection tool developed for key populations in Papua New Guinea

**Fig. 3:**
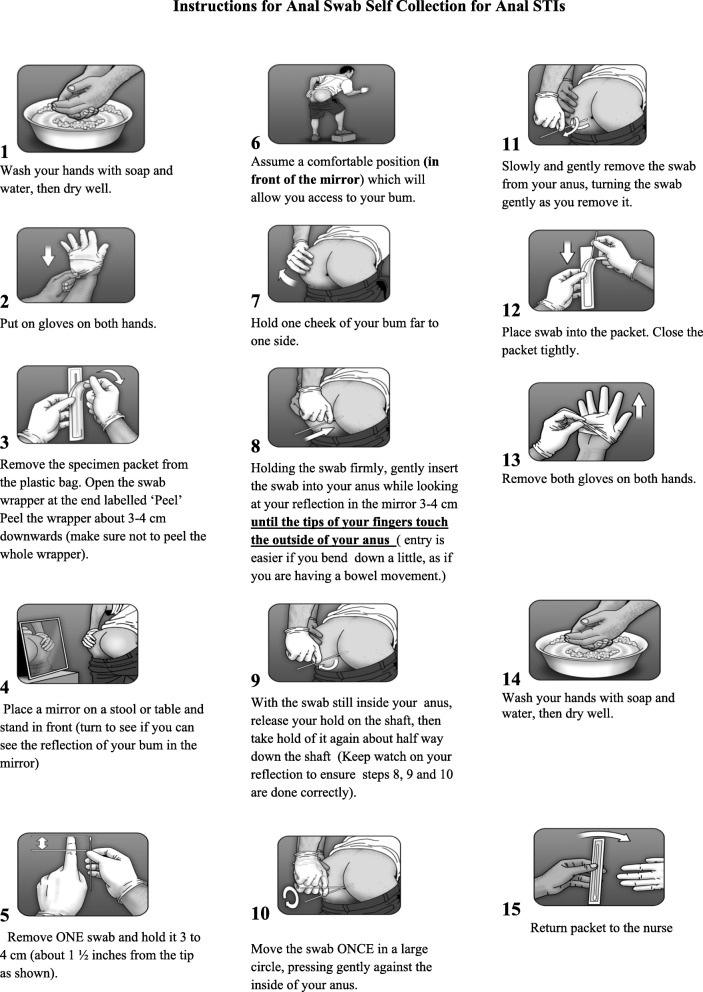
English version of the anorectal self-collection tool developed for key populations in Papua New Guinea

During focus groups, participants identified six changes that were required to modify the previously piloted and published instruction document. These are outlined in detail below. Furthermore, two language versions of the illustrated tool were produced – one in *Tok Pisin* (Fig. 2) and the other in English (Fig. 3). More significant changes were required for the *Tok Pisin* document (Fig. 2) than for the English (Fig. 3); participants agreed that only a few words would need simplification if people could read English. Findings on what changes needed to occur were similar across all key populations and study sites involved in this study.

### Finding 1: Identifying local terminology for ‘STIs’ and ‘anorectal STIs’

The original illustrated tool [9] assumes that participants understand that an anorectal sample can be collected and tested for a specific pathogen or biological characteristic. In PNG, general knowledge about STIs is relatively poor. All STI management is syndromic and anorectal examinations are not routine, even for people in key populations for whom anorectal STIs pose the greatest risk. In order to help research participants understand and localise terminology to explain the process of self-collected anorectal specimens, we identified locally relevant phrases to describe STIs first and then moved to the term for anorectal STIs. Anorectal STIs are part of a larger discussion on STIs, and it was appropriate to have more general conversations about STIs before introducing the more sensitive issues related to transmission of anorectal STIs.

Describing STIs was relatively straight forward, with focus group participants suggesting a translation of ‘*sik bilong koap*’ (Lit: infection from sex). Older terms originally used in PNG’s HIV and STI response, such as ‘*sik nogut*’ (Lit: bad sicknesses; usually associated with sex because of its immoral nature), were outright rejected by this population.

When discussing the terminology for anorectal STIs, participants decided that a more general phrase – ‘*sik bilong koap kain kain rot*’ (Lit: infection from sex in all different kinds of places) – would be most useful when health staff start conversations about testing for STIs, particularly anorectal STIs. This alluded to the development of STIs in a number of different places where people have sex – i.e. vagina, anus and mouth – and implies an offer for STI testing in two of the places where STIs can develop. For the *Tok Pisin* illustration, it was decided the term ‘*sik bilong as*’ (Lit: sickness of the anus) would be used.

### Finding 2: Identifying local terminology for the ‘anus’

In the original illustration in Fig. 1, the word ‘anus’ is referred to in stages 7–10 of the illustrated tool. The most anatomically correct description for the anus in *Tok Pisin,* ‘*rot bilong pekpek’* literally translates to ‘the passage belonging to faeces’. Research participants decided that this term was most appropriate to describe where self-collated swab specimens were to be taken from, and is used in stages 8, 9 and 11 of the newly adapted tool (Fig. 2).

### Finding 3: Identifying local terminology for ‘anal sex’

Although ‘anal sex’ is not a term used in the original illustrated tool, it is an important part of the discourse operating around the need for this tool. Research participants felt strongly that guidance ought to be offered to health workers and other individuals involved in any study on anorectal STIs to use correct terms for anal sex when explaining this specimen collection process and why anorectal STI testing is needed. This would avoid any embarrassment, and is an important aspect of enhancing cultural appropriateness in PNG.

Discussions about appropriate local terminology to describe anal sex were animated and lively. A number of different phrases for anal sex were identified by research participants for use with different audiences. After analysis of the focus group discussions, key populations decided to use ‘*koap long ass’* (Lit: anal sex), which was perceived as most appropriate as it was direct but less explicit than alternative options.

Focus group participants felt that sex workers, and other key populations, ‘must speak directly’ about anal sex, and ‘must listen to direct language’. Female sex workers used the terms far more explicit sexual language such as ‘*koap long ass*’ (Lit: anal sex) and ‘*koapim ass*’ (Lit: penetrate the anus) when talking with their peers. Although explicit, the use of the word ‘*long*’ makes the description of sex less offensive. Men who have sex with men, and transgender women used similar terms like ‘*koapim’* (Lit: penetrate) or ‘*koapim ass’* (Lit: penetrate the anus). They spoke directly, but usually in terms of their own sexual experience rather than offering ways to describe anal sex. For example, when differentiating oral from anal sex, one man said: ‘*mi save putim [kok] long maus or putim, long sas*’ (Lit: I usually put [my penis] in the mouth or the anus), or as one transgender woman said, ‘*kisim long ass hul bilong mi*’ (Lit: I take it in my anus).

However, all participants cautioned the need to use ‘less direct’ language with individuals who are not members of key populations. One female sex worker in Goroka stated that PNG is a Christian country, and ‘women who attend church’ – and, by inference, who are not sex workers – would deem the use of the explicit terms described above as inappropriate. For this reason, a politer term such as ‘*bungim bodi*’ (Lit: joining bodies) was perceived as being more appropriate for the general population, even though this term is ambiguous and does not relate to any specific sexual behaviour.

### Finding 4: Localising terms such as ‘specimen bottle’, ‘specimen packet’ and ‘swab’

In the original tool (see Fig. 1), specimen jars and PreservCyt solution are described in stages 2, 11, 12, 13 and 15. The sample collection routine utilised in PNG does not require these collection tools as they are used for HPV and not for Chlamydia and Neisseria Gonorrhoea which we were testing for. Instead, we use swab collection tubes. As such, as illustrated in stages 3, 12 and 15 of Figs. 2 and 3, research participants refer to the swab, and the plastic wrapper it was in, as the ‘specimen packet’ (swab collection tool) (i.e. the packet in which will hold your specimen).

The choice of words for these terms in *Tok Pisin* is challenging. Although *Tok Pisin* is not technically an evolving language, English words are frequently adopted and simplified to sound as if they are *Tok Pisin*. This has important implications for describing biomedical commodities like a ‘specimen’ or ‘swab’, and these required considerable effort to interpret for local use. While reading the English phrase “remove the specimen bottle” (Stage 2, Fig. 1), one of the senior and more educated female sex workers asked her peers, “How can we say that word [specimen] in *Tok Pisin*?” Even as the women tried to describe it in *Tok Pisin*, another said, “I’m still confused!”.

Focus group participants decided to keep the English word ‘specimen’ as the translation was too difficult, and ‘plastic’ was used to refer to the plastic packet. Similarly, it was difficult to translate the word ‘swab’ into *Tok Pisin*. Initially, because participants said that the swab looked like a ‘large ear bud’, participants decided to refer to it as *stik* (stick), but this is the same term that can be used to describe a straw. After further consultation, because of this ambiguity, participants recommended using the English term ‘swab’ in the *Tok Pisin* illustration. These are examples of how English words are readily adapted into *Tok Pisin* descriptions, sentences and instructions.

Other discussion arose around how to translate ‘open the swab wrapper’ in the original tool into *Tok Pisin*. Much talk occurred as to whether in language you would *brukim* (Lit: break) or *opim* (Lit: open) the packet. Rather than relying on literal translation, the term *brukim* was preferred in the final *Tok Pisin* version (Fig. 2, Stage 3).

### Finding 5: Describing a measurement of 3–4 cm

One of the most important aspects of the specimen self-collection procedure relates to a measurement of 3–4 cm, which is referred to in stages 4 and 7 of the tool in Fig. 1. Identifying the terminology to describe a measurement of 3-4 cm in *Tok Pisin* is difficult. With little formal education and the way in which *Tok Pisin* is used, measurements of time and distance are not unanimous with the specific way they are employed in the English language.

Participants noted the need to find a different way of describing how far the swab should be inserted into the anus. The answer evolved from a conversation about how research participants would measure the amount of water needed to cover the rice in a cooking pot before putting it on the stove or fire to cook. The pointer finger is frequently used, and the water measurement is marked at the knuckle, which equates to 3–4 cm. In *Tok Pisin*, this is referred to as ‘*makim long antap namel long pointa pinga’* (Lit: Just above the knuckle on the pointer finger)*,* which is the terminology used in stage 5 of Fig. 2. It was assumed that if individuals can read English, then they would understand a measurement of 3–4 cm, so this was left unchanged in Fig. 3. These changes also had obvious implications for the redesign of illustrations used in the adapted instruction document.

### Finding 6: Localising the illustrations

Focus group participants perceived the illustrations as an important way to facilitate understanding of this biomedical procedure. As one of the female sex workers stated, “these pictures, you will give us them to make it easy, won’t you?” It was agreed that the illustrations would be shown to the participants by clinicians, and would also be strategically placed in the clinic bathrooms where specimens would be collected.

Whilst the men and transgender women involved in the focus groups described the illustrations as ‘nice’, ‘good’ and ‘easy for us to follow’, they felt strongly that the illustrations should reflect PNG. They said, ‘*pitim piksa bilong yumi yet*’ (Lit: Put a picture of us). In stark contrast to many Papua New Guinean men, the muscular, slim, gym-going gay man in the original illustration was Caucasian and notably absent of any body hair.

Based on feedback during the focus groups, a number of adaptions were made to localise the illustration. These included the design of a new male PNG character with a fuller body shape, larger hands, generic clothing, and appropriate body and head hair and skin tone. In locations where sample collections would occur, including clinics, there is no running tap water, so a water basin was included instead. The ruler was replaced with the finger to illustrate the measurement issues described above.

The female sex worker focus group participants were of the opinion that two separate illustrations – one for women and one for men – was not necessary: ‘*Em inap tupela wankain*’ (Lit: It’s ok, it’s the same). They explained they were familiar with key population outreach work, and at ease with men who have sex with men and transgender women. However, should this tool be used more widely outside these population, these participants felt that a female character should also be depicted in the illustration.

## Discussion

It is widely recognised that health promotion materials must be culturally appropriate to be effective with different populations and cultural groups [20–22]. There is an increasing body of literature now examining the cultural appropriateness of instructions, information and protocols used during biomedical interventions [18, 23–27]. This is perceived as important to enhance the uptake and therefore impact of health interventions. To date in PNG and the Asia Pacific Region, limited research has been conducted in partnership with local populations to understand how biomedical procedures and instruments for anorectal testing can be modified to optimise acceptability and uptake of new interventions in socio-culturally diverse settings.

This paper reports on the development of a culturally appropriate tool that could be used by key populations for self-collection of anorectal specimens in PNG. It was necessary to ensure that the tool illustrated correct collection procedures that fitted the socio-cultural contexts of the populations who would use this guide. This is the first tool for self-collection we are aware of that has been designed by and for key populations in the Pacific. Furthermore, these findings have informed the inclusion of socio-culturally appropriate anorectal STI testing in a large bio-behavioural survey to be used among key populations in PNG for the first time [14–16].

These tools were piloted in the first three study sites of this bio-behavioural survey. As documented elsewhere [14–16], there were high rates of uptake of self-collection of anorectal samples for STI testing among key populations, as well as high yields of STI detected and valid results across all three sites. This illustrated that key populations participating in the bio-behavioural survey were able to correctly and effectively collect an anorectal specimen using these tools, and that no further no modifications were required.

By designing the illustrated tool in Figs. 2 and 3 in consultation with key populations in PNG, we can be sure that the explanation of the procedures outlined in Fig. 1 is clear, and fits with local constructions and terminology, and that people from these populations will identify with the visual design of the document. Of equal importance, the tool increases the probability of both the correct sample being collected and a valid test result being generated. By working closely with key populations to interpret and translate terms, phrases and instructions, we have recreated a specimen self-collection process that is acceptable to the population in need of anorectal STI testing and produced an illustrated specimen collection tool that is localised, appropriate, and relevant. By drawing on their own experiences of anal sex and anal STIs, and the marginalised social, cultural and religious contexts in which anal sex is practised, perceived and discussed, the research participants pointed to the necessity of tailoring dialogue used with the tool to ensure it is used ‘safely’ – with key populations, as well as other community members who might practice anal sex in their relationships.

Focus group narratives highlighted that localising this anorectal specimen self-collection procedure for use in this cultural setting was not straight forward. Understanding the populations for whom the instructions were being developed – in terms of the language they use, and varying levels of literacy – was required to enhance comprehension and interpretation of biomedical terminology. This became more evident as we progressed through the work. Findings illustrated the need to ensure linguistic accuracy and relevance while at the same time the need for a deeper cultural understanding of how language operates and information is transmitted within different social interactions.

There is persistent interest in understanding how to tailor health information and biomedical procedures, so these are more appropriate for culturally diverse populations [18, 28–30]. A seminal review on achieving cultural appropriateness for health materials outlines important dimensions of ensuring adequate cultural adaptation of health information, including *linguistic*, *socio-cultural*, *peripheral*, and *constituent-involving strategies* [21]. These were integral to the work undertaken in this study.

*Linguistic strategies* – making health communication literature and material available in the dominant or primary language [21] – were achieved through processes of translation and interpretation. In order to interpret meaning and comprehension effectively, *socio-cultural strategies* – ensuring a focus on understanding the ways that language operates, including the values, morals and cultural norms of communities the language imbues [21] – were implemented simultaneously. *Peripheral strategies* – ensuring a focus on the visual identity of documentation, including colours schemes, layout and characterisation [21] – were used to ensure that PNG-centred characters and scenarios were the focus of illustrations. This will enable key populations in PNG to realise that this procedure is relevant and important to them. The inclusion of a Papua New Guinean character was critical, and, for the populations involved in anorectal STI testing, research participants felt it was adequate to have a man. Finally, c*onstituent-involving strategies* – otherwise known as stakeholder or community engagement [21] – were central to the research design. The research experience highlighted that having a working knowledge of local language was not sufficient to succeed with the localisation of these sensitive procedures. Without investing time and energy to work in partnership with these members of key populations, we would not have understood the contexts in which terms are used, and the deeper meanings that terms encompass in different social interactions.

The terminology decided upon by the study participants for the illustrated guides suggests that there has been some progress in the discourses around HIV and that people are using more accurate, clear, and less stigmatising terms. It may also be the case that this is attributable to a different population who is more sexually liberated. Therefore, should the need arise to use this tool with other populations in PNG, particularly women who are not engaged in sex work, it would be advisable to revisit the illustrated tool, including the language used and the images chosen. For such women, it may not be appropriate to see a man depicted in the illustration, as it was acceptable to female sex workers.

## Conclusions

The development of this culturally appropriate illustrated tool for the self-collection of anorectal specimens for STI testing is the first of its kind in PNG. This process did not just involve literal translation of each word into *Tok Pisin*. Instead, by working in partnership with members of key populations at highest risk of anorectal STIs, the development of this tool required adequate local interpretation to ensure clear clinical instructions that could be followed, without isolating, offending or ostracising the people using the illustrated tool. Finding the best fit for words, phrases and descriptions took time and was an iterative process. This research process provides a case study of what can be required to ensure the cultural targeting of public health materials, working with marginalised communities to localise biomedical and clinical information, in ways that are respectful and empowering. This exercise has educated us as to how public health professionals could better frame talk about all sexual health issues among these populations.

## Abbreviations

HIV: human immunodeficiency virus
PNG: Papua New Guinea
STI: sexually transmitted infection
